# Changes in the Phenotype of Winter Wheat Varieties Released Between 1920 and 2016 in Response to In-Furrow Fertilizer: Biomass Allocation, Yield, and Grain Protein Concentration

**DOI:** 10.3389/fpls.2019.01786

**Published:** 2020-01-30

**Authors:** Rafael E. Maeoka, Victor O. Sadras, Ignacio A. Ciampitti, Dorivar R. Diaz, Allan K. Fritz, Romulo P. Lollato

**Affiliations:** ^1^ Department of Agronomy, Kansas State University, Manhattan, KS, United States; ^2^ South Australian Research and Development Institute, Adelaide, SA, Australia; ^3^ School of Agriculture, Food and Wine, The University of Adelaide, Adelaide, SA, Australia

**Keywords:** *Triticum aestivum* L., genetic progress, yield components, chronological change, biomass partitioning, harvest index, in-furrow fertilize

## Abstract

Plant breeding has increased the yield of winter wheat (*Triticum aestivum* L.) over decades, and the rate of genetic gain has been faster under high fertility in some countries. However, this response is not universal, and limited information exists on the physiological traits underlying the interaction between varieties and fertilization. Thus, our objectives were to identify the key shifts in crop phenotype in response to selection for yield and quality, and to determine whether historical and modern winter wheat varieties respond differently to in-furrow fertilizer. Factorial field experiments combined eight winter wheat varieties released between 1920 and 2016, and two fertilizer practices [control *versus* 112 kg ha^-1^ in-furrow 12 -40-0-10-1 (N-P-K-S-Zn)] in four Kansas environments. Grain yield and grain N-removal increased nonlinearly with year of release, with greater increases between 1966 and 2000. In-furrow fertilizer increased yield by ~300 kg ha^-1^ with no variety × fertility interaction. Grain protein concentration related negatively to yield, and the residuals of this relationship were unrelated to year of release. Yield increase was associated with changes in thermal time to critical growth stages, as modern varieties had shorter vegetative period and longer grain filling period. Yield gains also derived from more kernels m^-2^ resultant from more kernels head^-1^. Historical varieties were taller, had thinner stems, and allocated more biomass to the stem than semidwarf varieties. Yield gains resulted from increases in harvest index and not in biomass accumulation at grain filling and maturity, as shoot biomass was similar among varieties. The allometric exponent (i.e., the slope between log of organ biomass and log of shoot biomass) for grain increased with, and for leaves was unrelated to, year of release. The ability of modern varieties to allocate more biomass to the kernels coupled to an early maturity increased grain yield and grain N-removal over time. However, increases in grain yield were greater than increases in grain N-removal, reducing grain protein concentration in modern varieties.

## Introduction

Global wheat production often surpasses 750 Mt harvested from about 220 Mha, with an average yield of 3.4 Mg ha^-1^ (FAOSTAT, 2018). The development of semidwarf wheat varieties (Evenson, 2003) coupled with N fertilizer was responsible for large proportion of the yield advances over decades (Bell et al., 1995). The successful introduction of dwarfing genes carrying the alleles Rht1-B1b (Peng et al., 1999) allowed for plants with reduced height, greater response to fertilizer, and higher yields (Evenson, 2003). For irrigated spring wheat in Mexico, genetic improvement accounted for 28% and increased use of N fertilizers for 48% of the yield improvement between 1968 and 1990 (Bell et al., 1995). For dryland winter wheat in Kansas (U.S.) between 1977 and 2006, these estimates are 79% and 21%, respectively (Nalley et al., 2008).

Comparison of wheat varieties released during different historical eras returned rates of genetic gains from 0.3% to 1.1% (Austin et al., 1989; Sayre et al., 1997; Brancourt-Humel et al., 2003; Battenfield et al., 2013; Fischer et al., 2014). However, some studies showed that rates of yield gain can differ over time, and have typically decreased in recent years. For instance, genetic gain greater than 0.5% yr^-1^ during the 1960s to 2000s period was reported in the U.S., Australia, and Chile (Fufa et al., 2005; Sadras and Lawson, 2011; Del Pozo et al., 2014). Meanwhile, the genetic gain in wheat decreased or was nonsignificant in recent years in Spain, Brazil, and Argentina (Acreche et al., 2008; Beche et al., 2014; Lo Valvo et al., 2017).

Wheat yield gain is often associated with improved harvest index, kernels m^-2^, kernels per head, reduced plant height, shoot biomass and kernel weight (Brancourt-Humel et al., 2003; Zhou et al., 2007; Fischer and Edmeades, 2010; Sadras and Lawson, 2011; Sanchez-Garcia et al., 2013; Beche et al., 2014; Wu et al., 2014; Aisawi et al., 2015; Lo Valvo et al., 2017). More recently, genetic gain in yield correlated with shoot biomass at maturity in some breeding programs (Donmez et al., 2001; Shearman et al., 2005; Xiao et al., 2012; Beche et al., 2014; Wu et al., 2014) and agronomic studies across different varieties (de Oliveira Silva et al., 2019). However, previous research has not evaluated the dynamics of biomass accumulation and partitioning during the growing season of historical versus modern varieties (Pampana et al., 2007).

The rates of genetic gain are often greater in well-fertilized, well-watered crops than in their counterparts with water and/or nutrient deficiencies (Austin et al., 1980; Slafer and Andrade, 1989; Brancourt-Humel et al., 2003; De Vita et al., 2007; Giunta et al., 2007; Barraclough et al., 2010; Tian et al., 2011; Gizzi and Gambin, 2016; Wang et al., 2017). In-furrow fertilization with nitrogen, phosphorus, sulfur, and zinc can improve early-season wheat tillering, biomass production, and yield (Rodríguez et al., 1998; Rodríguez et al., 1999; Valle et al., 2009; Lollato et al., 2013). Nitrogen can increase grain yield (May et al., 2008; Grant et al., 2016) through kernels head^-1^ (Asif et al., 2012), heads m^-2^, and kernels m^-2^ (Marino et al., 2009). Phosphorus improves plant leaf area (Rodríguez et al., 1998) and tillering (Sato et al., 1996). Sulfur can increase grain yield and protein concentration (Tao et al., 2018), and Zn can increase pollen viability (Nautiyal et al., 2011). The combination of improved yield potential and management increased wheat yield worldwide; however, limited information exists on the changes in biomass accumulation and partitioning and on the interaction between historical and modern wheat varieties and in-furrow fertilization. Thus, our objectives were to: (i) determine grain yield increase due to genetic improvement of wheat adapted to Kansas, USA, by identifying the underlying changes in phenology, morphological, and physiological traits; and (ii) quantify how genotypes released in different eras respond to in-furrow fertilizer.

## Materials and Methods

### Site and Experiment Description

Field experiments were conducted in four environments resulting from the combination of two seasons and two locations in Kansas. In 2016-2017, experiments were established on a Belvue silt loam (coarse-silty, mixed, superactive, nonacid, mesic Typic Udifluvents) in Ashland Bottoms (39°08’37.8”N, 96°37’59.8”W, elevation 315 m) and on a Crete silt loam (fine, smectitic Pachic Udertic Argiustolls) in Belleville (39°48’54.1”N 97°40’16.7”W, elevation 469 m). In 2017–2018, experiments were conducted on an Ost loam (fine-loamy, mixed, superactive, mesic Udic Argiustolls) near Hutchinson (37°55’52.4”N 98°01’47.8”W, elevation 471 m) and again in Belleville.

Eight hard red winter wheat varieties released between 1920 and 2016 (**Supplementary Table 1**) were combined factorially with two fertilization treatments. The experimental design was a split-plot with four replications, with whole plots arranged as randomized complete block design and subplots completely randomized within whole plots. Varieties were assigned to plots and fertilizer treatment to subplots. The varieties were: “Kharkof,” released in 1920; “Scout 66” (1966); “Karl 92” (1992); “Jagger” (1994); “Jagalene” (2001); “Fuller” (2006); “KanMark” (2014); and “Larry” (2016). Varieties were selected based on large adoption by growers in the period following their release. Kharkof and Scout 66 carry the alleles Rht1-B1a-Tall and will hereafter be referred to as “tall varieties”; the remaining varieties carry the alleles Rht1-B1b-Short and will be referred to as “semidwarf varieties.” Due to seed germination issues, we excluded the data from Jagger during the first year. Fertilizer treatments were (i) control and (ii) 112 kg ha^-1^ in-furrow 12-40-00-10-1 (N-P-K-S-Zn) fertilizer, for a total application of 13, 45, 11, and 1 kg ha^-1^ of N, P_2_O_5_, S, and Zn. During the growing season, the entire experiment received the same amount of N fertilizer (see section “2.2. Agronomic management” for details) so that the only difference between treatments was the presence/absence of in-furrow fertilizer. The control treatment followed current soil fertility recommendations for P as per the nutrient “sufficiency” approach (Leikam et al., 2003), in which no in-furrow fertilizer was applied as the study locations had Mehlich-3 P above 25 mg kg^-1^ (**Table 1**). On the fertilization treatment, in-furrow fertilizer was applied at sowing through the drill with the seed.

**Table 1:** Initial soil pH in water, extractable P, K, Ca, Mg, Na, SO_4_-S, Zn, cation exchange capacity (CEC), organic matter (O.M.), and NO_3_-N for the 0–15 and 15–60 cm soil layers at Ashland Bottoms, Belleville and Hutchinson, KS.

Year	Location	fDepth (cm)	pH	P (mg kg^-1^)	K (mg kg^-1^)	Ca (mg kg^-1^)	Mg (mg kg^-1^)	Na (mg kg^-1^)	SO_4_-S (mg kg^-1^)	Zn (mg kg^-1^)	CEC (Meq 100g^-1^)	O.M. (g kg^-1^)	NO_3_-N (mg kg^-1^)	Applied N (kg ha^-1^)
2016-2017	Ashland B.	0 –15	6.0	41	190	975	105	13	1.7	0.3	10	13	3.6	105
		15 – 60	6.9	11	90	1,375	125	12	3.7	0.4	8	8	3.5	
	Belleville	0 – 15	5.9	42	474	1,532	202	13	2.9	1.5	21	30	4.5	158
		15 – 60	5.9	12	224	2,005	245	18	2.5	1.9	24	26	2.0	
2017-18	Hutchinson	0 – 15	6.0	77	218	1,886	238	11	3.4	2.3	20	24	6.2	63
		15 – 60	6.8	55	214	2,666	237	10	3.6	2.8	16	24	8.2	
	Belleville	0 – 15	5.6	42	400	1,727	228	10	3.0	0.9	22	28	8.9	67
		15 – 60	5.9	35	342	2,452	326	37	2.3	0.8	24	27	7.7	

Amount of inorganic N applied across the entire trial in each location during each growing season is also shown.

### Agronomic Management

Seeds were treated with insecticide and fungicide (15 ml 100 kg seed^-1^ of imidacloprid^1^ and with 0.74 ml 100 kg seed^-1^ of tebuconazole^2^) to control early-season insects and diseases. Wheat was sown 18 October 2016 at Ashland Bottoms, 3 October 2016 and 2 October 2017 at Belleville, and 19 October 2017 at Hutchinson. All crops followed a previous wheat crop and were conducted under conventional tillage with surface residue cover below 10%. Plots were sown with a commercial drill (Great Plains 606-NT drill) at a seeding rate of 67 kg ha^-1^ (approximately 2.1 million seeds ha^-1^). Subplots were 9.1-m long by 2.7-m wide, consisting of fourteen 0.19-m spaced rows. Half of the subplot (9.1 m x 1.33 m) was used for destructive sampling of biomass. The other half was used for nondestructive measurements (i.e., stem diameter and plant height), and harvested for yield.

Composite soil samples consisting of 15 individual soil cores were collected at two depths (0–15 cm and 15–60 cm) prior to sowing and analyzed for nutrient concentration (**Table 1**). Soil pH was analyzed through the procedure with water; soil P was measured through Mehlich-3; soil K, Ca, Mg, Na were measured through ammonium acetate extraction; soil S0_4_-S was measured through calcium phosphate extraction; soil Zn was analyzed through DTPA extraction; cation exchange capacity (CEC) was calculated through summation; soil organic matter was measured through loss of ignition; and NO_3_-N was measured through KCl extraction. Soil analyses were used to determine N fertilizer needs for all treatments using a yield goal of 6 Mg ha^-1^ (Leikam et al., 2003). This resulted in different total inorganic N amount in each site depending on the profile NO_3_-N content (i.e., 63 to 158 kg N ha^-1^, **Table 1**), but purposefully resulting in the same total N available to the wheat crop during the growing season. The entire trial was top-dressed with urea (46-0-0) in early spring (GS 31) under favorable weather to minimize N losses. Two foliar fungicide applications (i.e., 65.77 ml ha^-1^ of picoxystrobin^3^ at jointing [GS 31] and 89.15 ml ha^-1^ of picoxystrobin^1^ plus 35.63 ml ha^-1^ cyproconazole^4^ at anthesis [GS 65]) ensured that genetic resistance to fungal diseases was not a confounding factor. Herbicides were sprayed during fall of both growing seasons to ensure weeds were not a limiting factor. There was no significant insect pressure, so no insecticide was applied.

Plots were machine-harvested for grain yield on 22 June 2017 at Ashland Bottoms, 28 June 2017 and 24 June 2018 at Belleville, and 6 June 2018 at Hutchinson using a Hege 140 self-propelled small-plot combine. Grain moisture was measured at harvest and grain yield was corrected for 13% moisture content.

### Vegetative Development Evaluations

Phenological stages were determined using the Zadoks scale (Zadoks et al., 1974) when about 50% of the plants in the experimental unit achieved a particular stage. Shoot biomass was collected from the middle rows at tillering (GS 26), jointing (GS 31), anthesis (GS 65), soft dough stage of grain development (GS 85); and physiological maturity (GS 92) using an electric clipper (Gardena 8893-U, Gardena Co., Ulm, Germany). The sampled area was 0.76, 0.76, 0.38, 0.19, and 0.19 m^-2^, respectively, at an average stand of 185 plants m^-2^. Varieties differed in maturity and thus sampling occurred on different calendar days. Dry mass was determined after drying the samples at 65⁰C until constant weight. Shoot weight was determined at GS 26; stem and leaf weights were determined at GS 31; stem, leaf, and chaff weights were determined at GS 65; and stem, leaf, grain, and chaff weights were determined at GS 85 and GS 92. Plant parts were separated manually, except for grain and chaff, which were separated with a thresher (Wheat Head Thresher, PM Precision Machine Co. Inc., Lincoln, NE).

Stem diameter was measured at GS 85 using OriginCal IP54 digital caliper (Igaging, San Clement, CA) approximately 2.5 cm aboveground on the main stem of ten randomly selected plants per subplot. Plant height was measured at GS 92 from the soil surface until the tip of the awns of three plants per subplot. Yield components (harvest index, heads m^-2^, kernels head^-1^, kernels m^-2^, and individual kernel weight) were measured in the sample collected at physiological maturity. Grain protein concentration (g kg^-1^) was measured in the whole kernel from samples collected at harvest using near-infrared reflectance spectroscopy with a Perten DA 7250 (Perten Instruments Inc., Springfield, Illinois) and was reported on a 13% water basis. Grain N concentration was measured at GS 92 using the procedure of dry combustion (TruSpec CN, LECO Corporation, St. Joseph, MI, 2005). Grain-N removal was calculated as the product between grain yield and grain nitrogen concentration (Lollato et al., 2019a).

### Data Analysis

Two-way analyses of variance (ANOVA) were performed to determine significant difference among treatments using PROC GLIMMIX in SAS version 9.4 **Supplementary Table 2**. (SAS Institute, Cary, NC). To determine whether site-years could be combined, we performed an ANOVA on the residuals of the combined analysis considering year, location, variety, and fertility, and their interactions, as fixed effects. Year was a significant effect for both biomass (*p* < 0.05) and grain yield (*p* = 0.08); thus, we performed all remaining analysis across locations within year. Variety, fertility, and variety × fertility were fixed effects; and replication, sites, replication nested within site, and variety × replications nested within site were random effects. We used the LINES statement for pairwise comparisons.

To evaluate historical trends across the entire data set, we calculated trait deviation from the mean of each environment (Sadras and Lawson, 2011) and fitted seven models to the deviation data as a function of year of release (i.e., logarithm, logistic, piecewise, linear, quadratic, sigmoidal, and cubic). Models were fitted with SigmaPlot version 13.0 (Systat Software, San Jose, CA). The best model was selected using the Akaike information criterion (AIC) and the coefficient of determination (R^2^). We also considered the agronomic significance and interpretability of the models tested. We analyzed the residuals of these relationships for the fertilizer effect (Sadras and Moran, 2012). Because grain protein concentration is dependent on yield (Simmonds, 1995; Oury and Godin, 2007; Bogard et al., 2010), we first fitted a linear regression between deviations of grain protein and yield. Then, we analyzed the residuals of this relationship against year of variety release and fertilizer practice (Ortez et al., 2018).

Shoot biomass as affected by thermal time was first evaluated using the ANOVA procedure described above at each growth stage for whole plant biomass, and afterwards, for each individual plant component at each growth stage. Thermal time (growing degree days, GDD°C) was calculated as the sum of daily minimum and maximum temperature divided by 2 considering a base temperature of 0⁰C (Gallagher, 1979). Crop growth rate was calculated as the difference in shoot biomass between two successive samplings, divided by the intervening thermal time. Allometric relationships between biomass of plant organs (leaf, stem, chaff, and grain) and shoot were evaluated using standardized major axis (SMA) in through SMATR package (SMATR version 3; Warton et al., 2012) in R software (R development Core team, 2016). Plant organs and shoot biomass were log_10_ transformed prior to this analysis (Niklas, 2006), and time trends in allometric coefficients were evaluated by regression the slope of this relationship (logY = α logX) against year of variety release. Nonlinear models and historical trends were fitted with SigmaPlot version 13.0 (Systat Software, San Jose, CA).

We performed a final, comprehensive analysis across the entire data set using seven statistical procedures (stepwise, forward, backward, least angle regression (LAR), least squared shrinkage operator (LASSO), elastic net, and conditional inference trees) to assess the influence of all measured traits and environmental conditions on grain yield. Environmental conditions were calculated for different developmental windows (i.e., the entire cycle, the 30-d period prior to anthesis, and the grain filling period) and included average maximum and minimum temperatures, cumulative precipitation, cumulative solar radiation, and photothermal quotient (Fischer, 1985). All models were built in PROC GMSELECT in SAS version 9.4 (SAS Institute, Cary, NC) except for the conditional inference tree, which was built using the *partykit* package in R (R development Core team, 2016). Intermediate node and terminal node included a minimum of 10% of total observations. A sensitivity analysis allowed less observations to form nodes, but the model fit was improved in less than 10% so the most parsimonious model was selected.

## Results

### Weather Conditions

Seasonal precipitation ranged between 281 and 472 mm. Seasonal differences were more apparent during the fall and winter, with spring precipitation ranging between 169 and 262 mm at both growing seasons (**Table 2**). These conditions led to lower biomass in 2016-2017, precluding a combined analysis of the data. Despite lower seasonal total precipitation, favorable spring weather led to greater grain yield in 2017-2018.

**Table 2:** Cumulative precipitation (Precip.), average maximum (T_max_), and minimum temperatures (T_min_), cumulative solar radiation (R_s_) in (MJ m^-2^), and average photothermal quotient (PTQ) for each portion of growing season during 2016-2017 and 2017-2018 at Ashland Bottoms, Belleville, and Hutchinson, KS.

Year	Location	Precip.			T_min_			T_max_			R_s_			PTQ		
		Fall^a^ (mm)	Winter^b^ (mm)	Spring^c^ (mm)	Fall^a^ (⁰C)	Winter^b^ (⁰C)	Spring^c^ (⁰C)	Fall^a^ (⁰C)	Winter^b^ (⁰C)	Spring^c^ (⁰C)	Fall^a^ (MJ m^-2^)	Winter^b^ (MJ m^-2^)	Spring^c^ (MJ m^-2^)	Fall^a^ (MJ m^-2^ d^-1^ ⁰C^-1^)	Winter^b^ (MJ m^-2^ d^-1^ ⁰C^-1^)	Spring^c^ (MJ m^-2^ d^-1^ ⁰C^-1^)
2016-2017	Ashland B.	99	146	227	2	−1	12	16	11	25	1,027	1,083	1,909	0.79	0.77	1.82
	Belleville	91	74	262	−1	−3	10	14	10	24	876	996	1,938	0.64	0.56	1.71
2017-2018	Hutchinson	52	60	169	0	−5	11	14	10	26	874	1,063	1,567	0.62	0.55	1.33
	Belleville	37	36	217	−3	−8	10	12	6	24	861	1,030	1,811	0.54	0.30	1.33
30-year mean	Ashland B.	119	87	318	1	−4	12	13	9	25	792	941	1,745	0.59	0.47	1.41
	Belleville	85	71	256	−1	−6	10	12	8	24	826	1,002	1,839	0.57	0.42	1.56
	Hutchinson	125	102	264	2	−3	10	15	9	23	764	786	1,396	0.57	0.41	1.16

a Fall encompasses October, November, and December.

b Winter encompasses January, February, and March.

c Spring encompasses the period between April 1^st^ and harvest. The 30-year mean of each variable for each location is also shown.

### Plant Height, Stem Diameter, and Phenology

**Figure 1:**
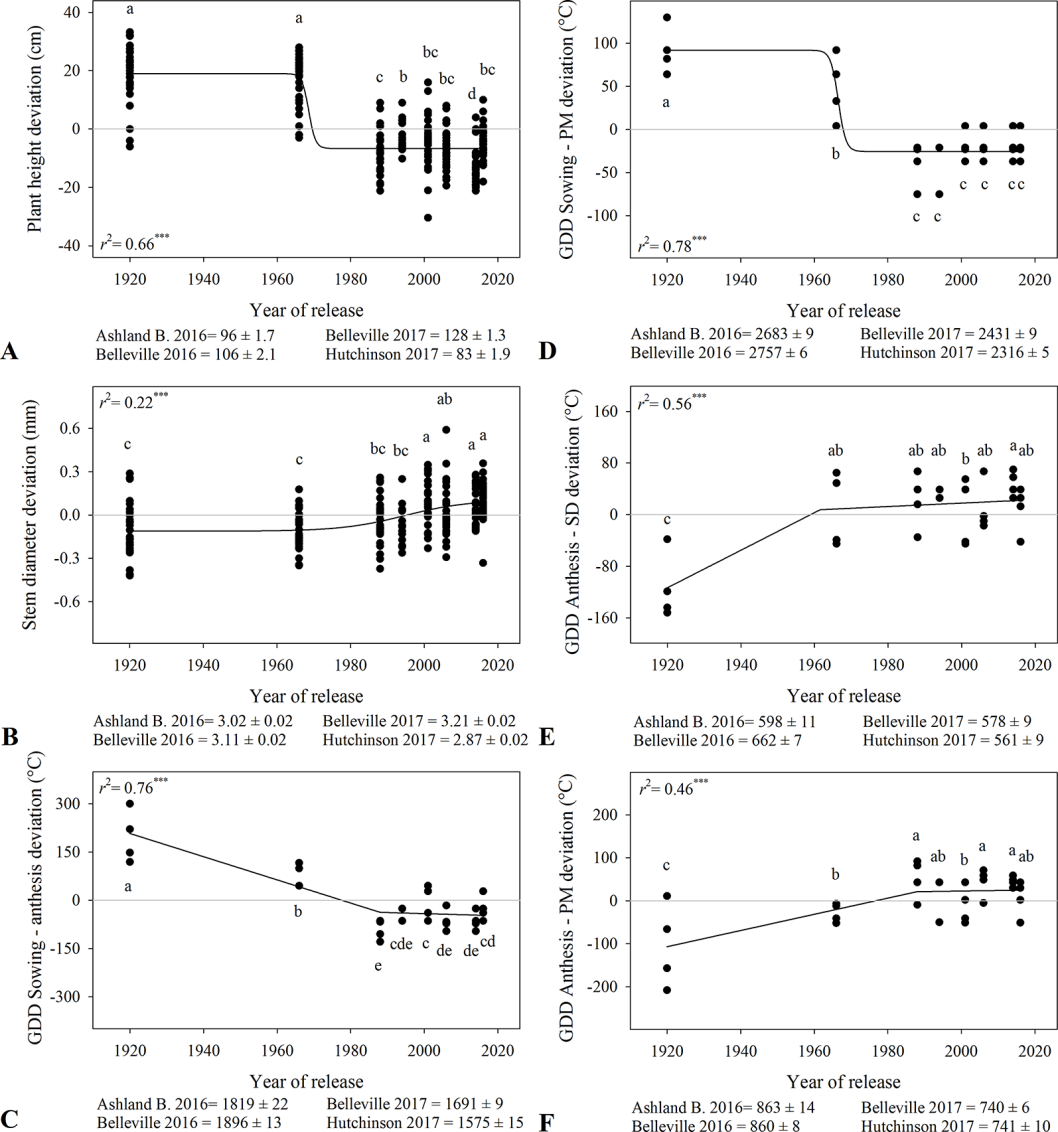
Relationship between year of release and plant height **(A)**, stem diameter **(B)**, and thermal time from sowing to anthesis **(C)**, sowing to physiological maturity **(D),** anthesis to soft dough **(E)**, and anthesis to physiological maturity **(F)**. Values correspond to the data of four site-years during two growing seasons (2016-2017 and 2017-2018). Mean for all varieties in each site and year. All models are significant at P < 0.0001. Varieties followed by the same letter are not statistically different at α = 0.05.

Plant height decreased over time with a steep change around ~1970s from 122 cm to 93-100 cm (**Figure 1A**, **Table 3**). Stem diameter ranged from 2.87 to 3.21 mm among locations and increased with year of release, particularly from 1960s to 2000s (**Figure 1B**). Time from sowing to anthesis and to physiological maturity decreased over time (**Figures 1C, D**), and modern varieties had a longer period from anthesis to physiological maturity (**Figure 1F**). The duration of the period between anthesis and physiological maturity associated linearly and positively with harvest index (*r* ^2^ > 0.14, data not shown), suggesting that the increase in wheat yield in modern varieties was partially explained by a longer grain fill. However, varieties released in the last 30 years showed minimal developmental changes (**Figure 1**).

**Table 3:** Grain yield, head number, head size, kernel number, kernel weight, harvest index (HI), plant height (PH), stem diameter, grain protein concentration (GPC) and grain volume weight (GVW) of wheat varieties released between 1920 and 2016, fertilizer treatment, and their interaction during the growing seasons 2016-2017 and 2017-2018.

Year	Variety	Year of release	Fertilizer	Grain Yield (Mg ha^-1^)	Nitrogen removal (kg ha^-1^)	GPC (g kg^-1^)	Head number (heads m^-2^)	Head size (kernels head^-1^)	Kernel number (kernels m^-2^)	Kernel weight (mg kernel^-1^)	HI	PH (cm)	Stem Diameter (mm)	GVW (kg m^-3^)
2016-2017	Kharkof	(1920)		1.7e	54d	135ab	813bc	11d	9383c	22.0b	0.13c	120a	2.88c	728e
	Scout 66	(1966)		2.4d	74c	133ab	931a	12d	10863c	26.7a	0.16c	123a	2.90c	751c
	Karl 92	(1988)		3.4c	103b	138a	854ab	14c	12221bc	26.8a	0.26b	93c	3.00b	744d
	Jagalene	(2001)		5.2a	142a	122c	755bcd	23ab	17075a	27.2a	0.34a	95bc	3.17a	770a
	Fuller	(2006)		4.5b	127a	131b	677d	23ab	15763a	27.9a	0.32ab	92c	3.16a	755bc
	KanMark	(2014)		5.2a	138a	119c	712cd	24a	17904a	26.4a	0.37a	87d	3.18a	776a
	Larry	(2016)		5.1ab	138a	121c	708cd	21b	15312ba	28.3a	0.36a	98b	3.18a	759b
			In-furrow	4.0A	114A	129	825A	17B	13946	26.1B	0.28	102	3.06	754B
			Control	3.8B	108B	128	733B	20A	14203	26.9A	0.28	100	3.07	756A
2017-2018	Kharkof	(1920)		3.9d	116d	150a	767a	17e	12852c	22.9c	0.39c	125a	3.00	723ab
	Scout 66	(1966)		4.9c	137c	142b	766a	19de	14501bc	27.4a	0.44bc	119a	3.00	712bc
	Karl 92	(1988)		5.5b	148bc	137bcd	731ab	21d	15322bc	27.3a	0.54a	100bc	3.04	731a
	Jagger	(1994)		5.9ab	155ab	139bcd	721ab	28bc	19982a	25.1b	0.50ab	104b	2.98	694de
	Jagalene	(2001)		6.3a	163a	134cde	660bc	28abc	18443a	27.3a	0.56a	103b	3.07	721ab
	Fuller	(2006)		5.5b	144bc	140bc	609c	26c	15657b	25.8b	0.52a	102b	3.02	681e
	KanMark	(2014)		6.2a	152ab	129e	698ab	30a	21041a	24.9b	0.52a	94c	3.08	718b
	Larry	(2016)		6.2a	166a	133de	666bc	29ab	19274a	26.2ab	0.55a	101bc	3.13	704cd
			In-furrow	5.8A	152Aa	137B	737A	24B	17237	25.4B	0.51	106	3.03	708B
			Control	5.4B	143Bb	139A	667B	26A	17031	26.3A	0.50	106	3.05	713A

Values followed by the same letter within growing season and treatment (lower case letters for varieties and capitalized letters for fertility) are not statistically different at α = 0.05.

Jagger was not included in the 2016-2017 growing season analysis.

Variety and fertilizer means were averaged across locations within growing season.

### Grain Yield, Grain-N Removal, and Grain Protein Concentration

**Figure 2:**
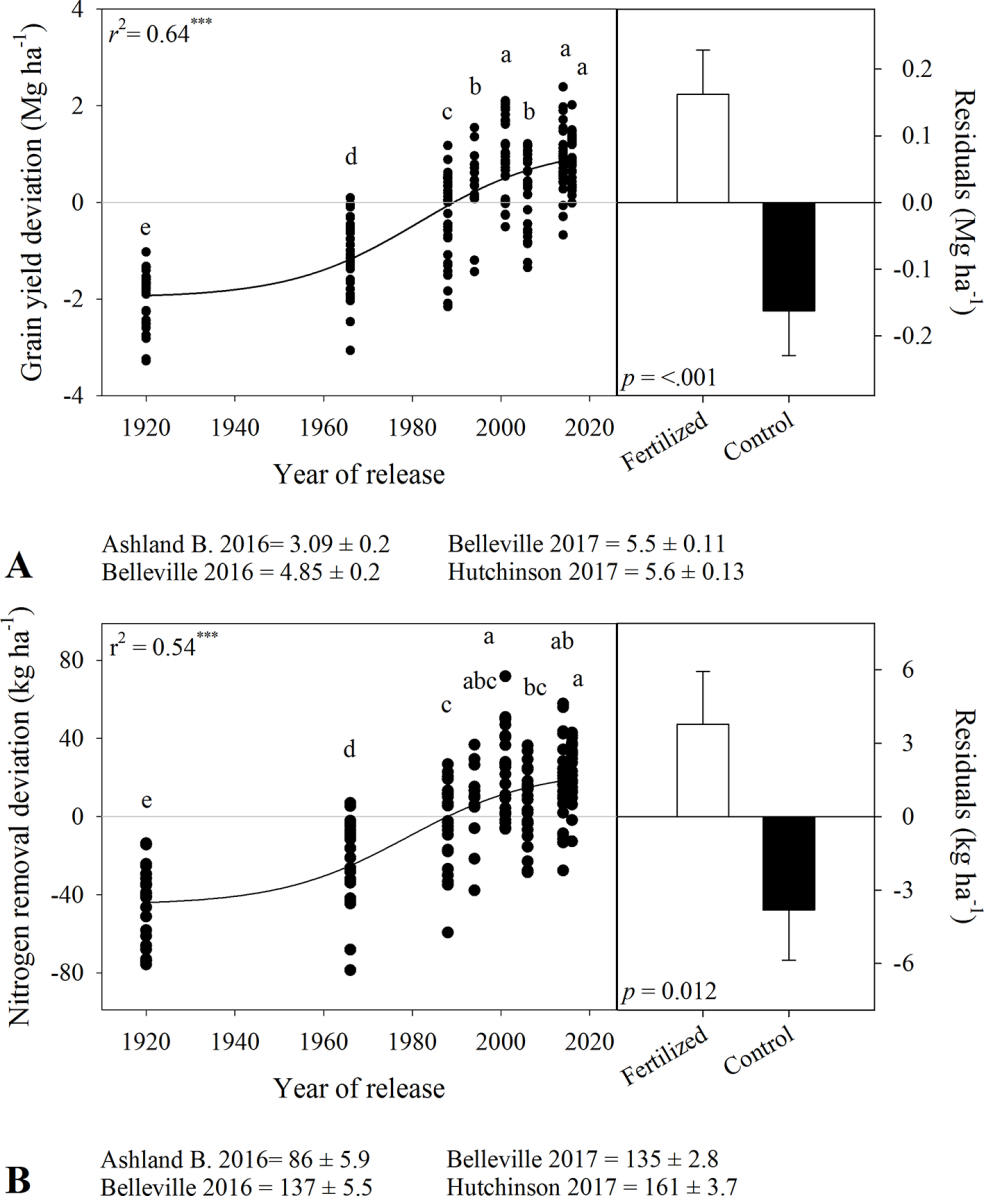
Relationship between year of release and **(A)** grain yield deviation and **(B)** grain nitrogen removal deviation. Comparison of the residuals of the regressions for in-furrow fertilizer treatment and control (bars show mean and standard error). Values correspond to the data of four site-years during two growing seasons (2016-2017 and 2017-2018). Mean for all varieties in each site and year. Both curves are significant at P < 0.0001. Varieties followed by the same letter are not statistically different at α = 0.05.

There were significant variety and fertility effects on wheat grain yield in both seasons, with no variety × fertility interaction (**Table 3**). Grain yield ranged from 1.7 to 4.9 Mg ha^-1^ for tall varieties and from 3.4 to 6.3 Mg ha^-1^ for semidwarf varieties. In-furrow fertilizer increased mean yield by 0.2 to 0.4 Mg ha^-1^ in relation to the control. Grain yield increased nonlinearly with year of release (**Figure 2A**), with three distinct rates. A low yield-gain period between 1920 and 1966 (17 kg ha^-1^ yr^-1^), followed by a steep yield gain between 1966 and 2000 (62 kg ha^-1^ yr^-1^), and a slower yield gain phase after 2000 (8 kg ha^-1^ yr^-1^).

There were significant variety and fertility effects on grain-N removal (**Table 3**). Grain-N removal increased from tall to semidwarf varieties (c.a., 64 to 130 kg ha^-1^ in 2016-2017 and 127 to 155 kg ha^-1^ in 2017-2018). In-furrow fertilizer increased grain-N removal by 6 to 9 kg ha^-1^. Similar to grain yield, grain-N removal deviation increased nonlinearly with year of release, with linear rates of 0.44, 1.28, and 0.11 kg ha^-1^ yr^-1^ for the aforementioned periods (**Figure 2B**).

Typically, tall varieties had greater grain protein concentration than the semidwarf varieties. In 2016-2017, there was a significant interaction between variety and fertility on grain protein concentration (**Table 3**) as most varieties increased grain protein concentration in response to in-furrow fertilizer except by the semidwarf varieties Fuller and KanMark (data not shown).

**Figure 3:**
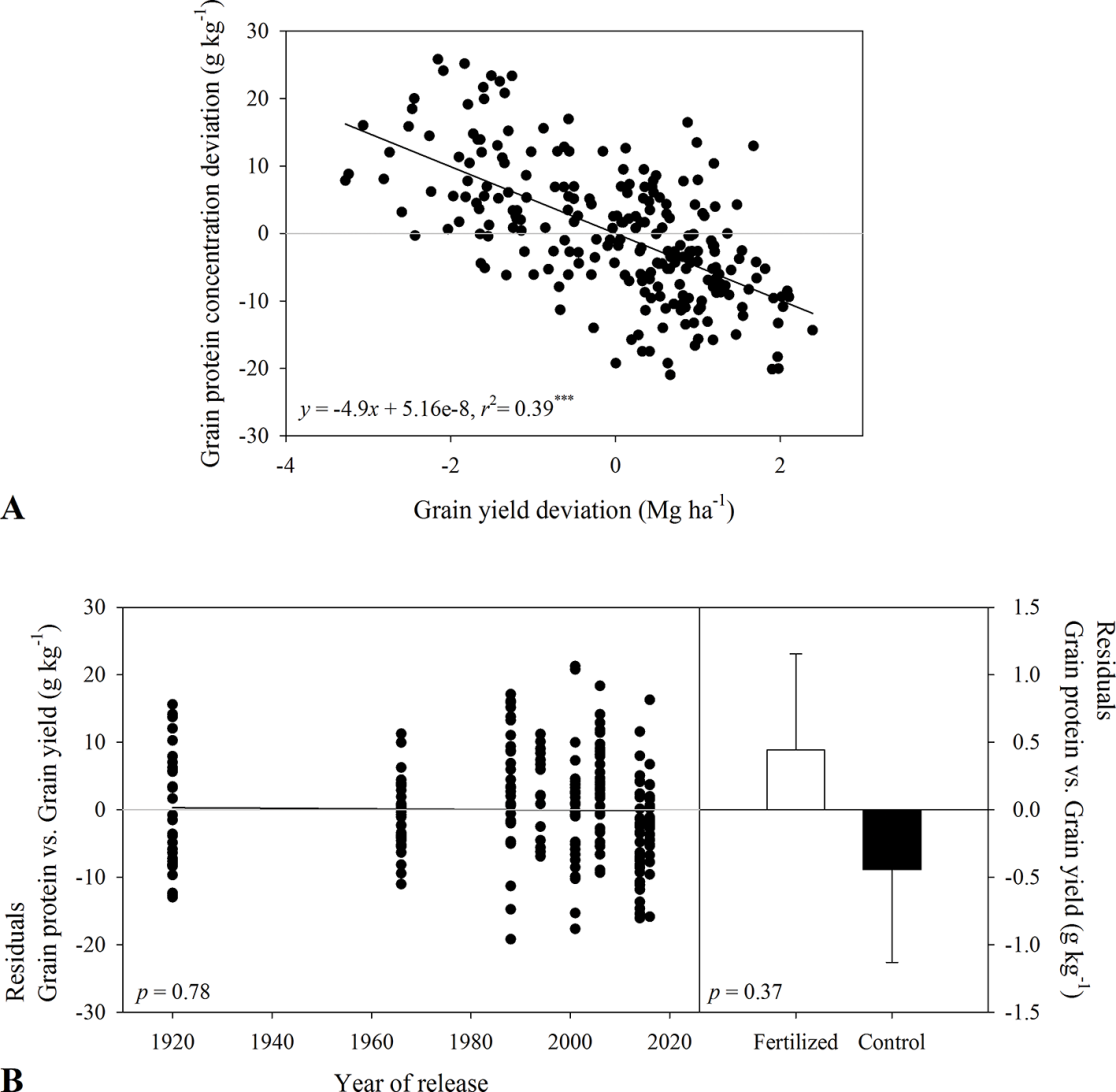
Relationship between **(A)** grain protein concentration deviation and grain yield deviation for varieties released between 1920 and 2016. Residuals of the regression **(B)** as affected by year of variety release or in-furrow fertilizer treatment and control (bars show mean and standard error). Values correspond to the data of four site-years during two growing seasons (2016-2017 and 2017-2018).

In 2017-2018, grain protein concentration in tall varieties was 142 to 150 g kg^-1^ compared to 129 to 140 g kg^-1^ in semidwarf varieties. In-furrow fertilizer decreased grain protein concentration (**Table 3**). Grain protein deviation declined linearly with grain yield deviation (**Figure 3A**), and the residuals of this relationship were unrelated to year of release (*p* > 0.37, **Figure 3B**).

### Yield Components

**Figure 4:**
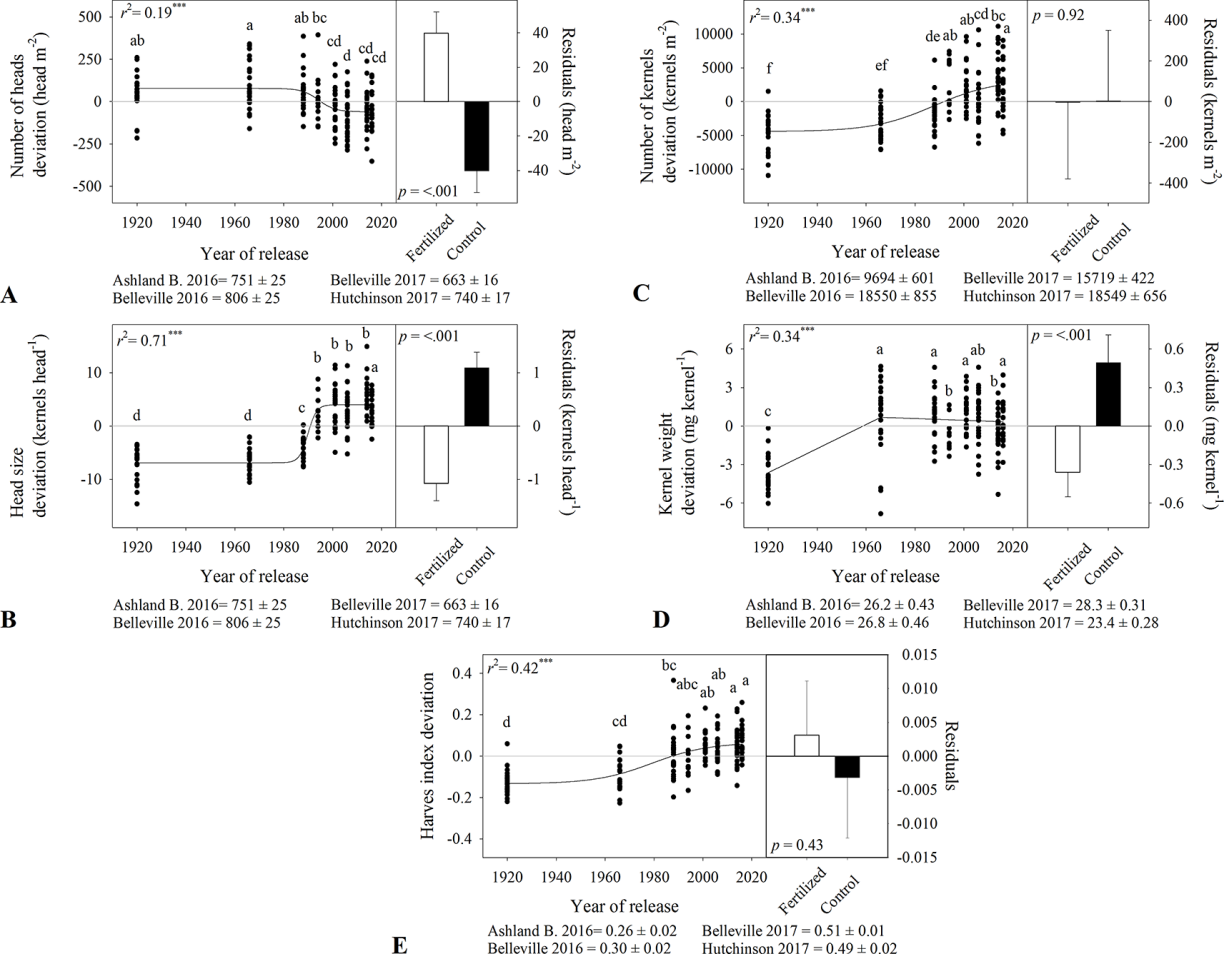
Relationship between the year of release and yield components deviation including **(A)** heads number, **(B)** head size, **(C)** kernel number, **(D)** kernel weight, and **(E)** harvest index. Comparison of the regression residuals for in-furrow fertilizer treatment and no control (mean and one standard error). Values correspond to the data of four site years during two growing seasons (2016-2017 and 2017-2018). Mean for all varieties in each site and year. All models are significant at P < 0.0001. Varieties followed by the same letter are not statistically different at α = 0.05.

There was a nonlinear relationship between heads m^-2^ and year of release, with modern varieties having fewer heads m^-2^ (greater differences between late 1980s until early 2000s, **Figure 4A**). Tall varieties had 872 and 767 heads m^-2^ while semidwarf varieties had 741 and 680 heads m^-2^ during 2016-2017 and 2017-2018 (**Table 3**). As heads m^-2^ decreased over time, kernels head^-1^ increased, from 12–18 kernels head^-1^ to 21–27 kernels head^-1^ with greater increases after 1980s (**Figure 4B** and **Table 3**). Due to the contrasting trends in heads m^-2^ and kernels head^-1^, the increase in kernels m^-2^ was slower but significant (**Figure 4C**). The tall variety Kharkof had the lowest kernels m^-2^ (i.e., 9,383 and 12,852 kernels m^-2^) while the semidwarf variety KanMark had the highest (i.e., 17,904 and 21,041 kernels m^-2^).

Kernel weight showed a bilinear relationship with year of release (**Figure 4D**), increasing at a higher rate until 1966 and remaining constant afterwards (**Table 3**). Harvest index increased nonlinearly over time and ranged from 0.26 to 0.51 among locations (**Figure 4E**). While harvest index increased from 0.15 to 0.33 in 2016-2017; differences were smaller in 2017-2018 (**Table 3**). Variety affected grain volume weight in both growing seasons, both with no consistent time trends (**Table 3**).

In-furrow fertilizer increased heads m^-2^ (**Figure 4A**) from 733 to 825 in 2016-2017, and from 667 to 737 heads m^-2^ in 2017-2018 (**Table 3**). However, it decreased kernels head^-1^ (**Figure 4B**) from 20 to 17 in 2016-2017 and from 26 to 24 in 2017-2018 (**Table 3**). Fertilizer had no effect on kernels m^-2^ (**Figure 4C**) and decreased kernel weight (**Figure 4D** and **Table 3**) from 26.9 to 26.1 mg kernel^-1^ in 2016-2017 and from 26.3 to 25.4 mg kernel^-1^ in 2017-2018. There were no differences in harvest index between the fertilizer practices (**Figure 4E**), and in-furrow fertilizer showed lower volume weight than control (**Table 3**).

### Shoot Biomass, Crop Growth Rate, and Biomass Allocation to Plant Components

There was no clear pattern in biomass among varieties early in the season (i.e. at GS 26 and 31), but tall varieties had greater shoot biomass than semidwarf ones at anthesis (861–1,087 g m^-2^ *versus* 658–888 g m^-2^) (**Table 4**). These differences related positively to thermal time from sowing to anthesis (*r* ^2^ = 0.42). However, these differences were not apparent (2017-2018) or were reversed (2016-2017) at soft dough (GS 85), when semidwarf varieties showed up to 30% greater biomass relative to tall varieties. There were no differences in biomass among varieties at maturity, and in-furrow fertilizer increased biomass irrespective of growth stage.

**Table 4:** Shoot biomass in different plant components (leaves, stem, chaff, grain) at Zadoks 26, 31, 65, 85, and 92 of wheat varieties released between 1920 and 2016, fertilizer treatment, and their interaction during the growing seasons 2016-2017 and 2017-2018. Variety and fertilizer means were averaged across locations within growing season.

Year	Variety (Year of release)	Fertilizer	GS 26	GS 31		GS 65			GS 85				GS 92			
			Leaves(g m^-2^)	Leaves (g m^-2^)	Stem (g m^-2^)	Leaves (g m^-2^)	Stem (g m^-2^)	Chaff (g m^-2^)	Leaves (g m^-2^)	Stem (g m^-2^)	Chaff (g m^-2^)	Grain (g m^-2^)	Leaves (g m^-2^)	Stem (g m^-2^)	Chaff (g m^-2^)	Grain (g m^-2^)
2016-2017	Kharkof (1920)		60.8a	158.3	88.3c	243.1a	749.9a	103.2c	190.1	798.3a	148.0c	185.7c	159.5a	797.2a	154.7c	209.1c
	Scout 66 (1966)		56.7a	156.3	128.9ab	224.0ab	707.2a	131.8b	171.4	757.1a	169.5c	227.3c	144.6ab	808.9a	196.2ab	285.5bc
	Karl 92 (1988)		58.6a	131.8	134.9a	184.2b	493.0d	134.5b	151.3	730.8a	238.5ab	365.6b	103.2d	589.5b	227.5a	331.2b
	Jagalene (2001)		54.0a	150.5	102.6bc	221.1ab	603.5b	154.6a	192.3	804.7a	247.7a	582.6a	132.7abcd	611.0b	210.5ab	464.6a
	Fuller (2006)		43.1b	134.8	113.3abc	190.7b	525.5cd	133.4b	164.9	805.9a	238.7ab	492.6a	109.2cd	581.8b	202.5ab	439.3a
	KanMark (2014)		43.4b	138.3	100.9c	185.1b	458.5d	143.5ab	168.9	600.9b	247.9a	538.8a	125.9bcd	512.8b	221.9ab	474.3a
	Larry (2016)		57.9a	154.7	96.9c	221.4ab	585.6bc	144.9ab	212.8	759.5a	216.1b	514.8a	133.7abc	587.1b	189.8bc	425.6a
		In-furrow	63.5A	163.9A	126.5A	228.2A	626.5A	140.3A	198.8A	807.6A	227.0A	443.9	140.9A	674.4A	204.5	365.0
		Control	43.4B	128.9B	92.3B	191.7B	551.6B	130.0B	158.9B	694.4B	203.4B	396.8	118.8B	607.9B	196.3	386.3
2017-2018	Kharkof (1920)		36.0	102.1	55.0bc	150.4	466.7a	201.1a	112.9	471.7a	181.3	308.2d	93.6	441.5a	184.3	292.3c
	Scout 66 (1966)		30.0	118.6	80.5a	187.5	498.9a	196.5ab	103.1	451.2a	188.5	408.6c	91.1	417.2a	201.0	397.8b
	Karl 92 (1988)		38.8	97.6	69.6ab	140.1	335.7b	149.3cd	84.9	327.7b	194.1	436.1bc	72.4	299.0bc	190.1	415.3b
	Jagger (1994)		35.2	103.5	66.7ab	171.7	340.0b	142.3d	98.3	355.1b	188.6	478.6ab	94.9	351.4b	196.9	491.4a
	Jagalene (2001)		36.4	104.8	60.2b	171.0	381.0b	162.3bcd	91.0	332.5b	172.9	488.9ab	84.3	312.3bc	187.4	495.2a
	Fuller (2006)		31.9	89.4	61.5b	158.6	327.5b	144.5cd	98.3	333.3b	189.7	433.6bc	85.9	306.8bc	199.7	406.0b
	KanMark (2014)		34.1	107.0	62.2b	192.8	330.4b	178.3abc	110.9	305.4b	209.0	511.8a	100.9	297.0c	210.5	519.9a
	Larry (2016)		33.8	95.7	42.3c	199.0	349.9b	173.6abcd	99.0	343.0b	186.9	494.4ab	90.5	325.4bc	189.5	496.9a
		In-furrow	41.5A	120.5A	79.3A	193.6A	416.9A	181.8A	112.8A	405.2A	203.3A	471.2A	97.5A	365.3A	201.2A	434.8
		Control	27.4B	84.2B	45.2B	149.2B	340.6B	155.2B	86.8B	324.8B	174.5B	418.8B	80.9B	322.4B	188.7B	443.9

Values followed by the same letter within growing season and treatment are not statistically different at α = 0.05.

Jagger was not included in the 2016-2017 growing season analysis.

Crop growth rate was low (c.a., 0.08 to 0.3 g m^-2°^C^-1^ day^-1^) between tillering and jointing, and increased to about 1.3–1.5 g m^-2°^C^-1^ day^-1^ between GS 31 and GS 65 (**Supplementary Table 2**). There were no differences among varieties early in the season, although in-furrow fertilizer increased growth rate. The growth rate in semidwarf varieties was as much as two times greater than in tall varieties from anthesis to soft dough in the first season (**Supplementary Table 3**), decreasing after soft dough.

There were no clear differences among varieties in their allocation of biomass toward leaves and stem early in the season (**Table 4**). However, at anthesis and soft dough tall varieties showed greater biomass in the stem relative to semidwarf varieties (c.a., 605 *versus* 430 g m^-2^, and 620 *versus* 518 g m^-2^). During 2016-2017, tall varieties also showed greater leaf biomass than semidwarf varieties at GS 65 and GS 92 (**Table 4**). Grain biomass at soft dough stage was greater in semidwarf varieties in both growing seasons, and dry matter partitioning to leaves and stem ceased at this stage regardless of year of release. Grain biomass at maturity in semidwarf varieties was as much as 470 g m^-2^ (2017-18) and no more than 345 g m^-2^ (2017-18) for tall varieties (**Table 4**). In-furrow fertilizer increased biomass irrespective of growth stage and plant component (**Table 4**); however, more biomass was allocated to vegetative tissues than to grain.

**Figure 5:**
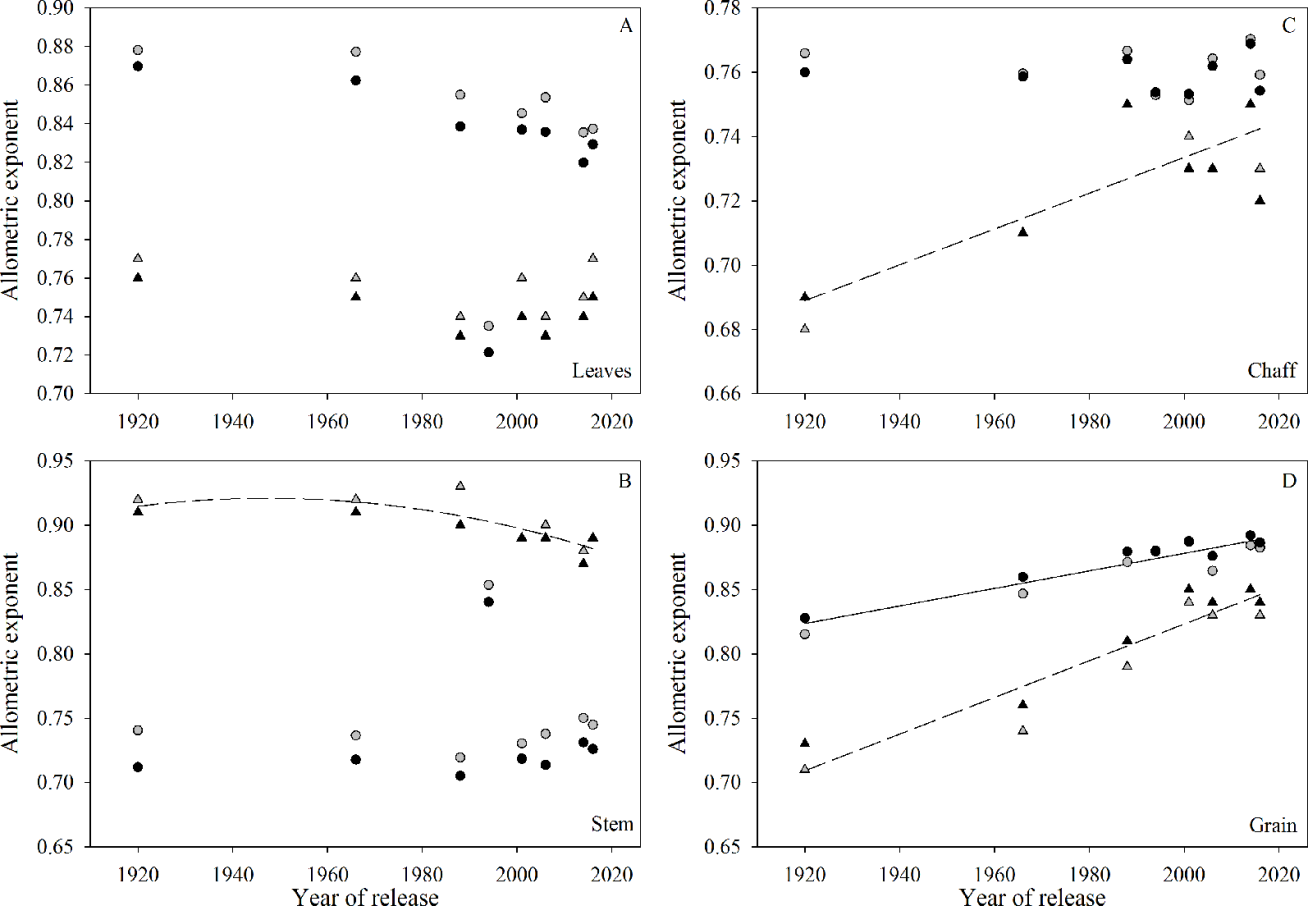
Relationship between allometric exponent (slope of log of plant organ biomass *versus* log of shoot biomass) and year of release for each plant organ: **(A)** leaves, **(B)** stem, **(C)** chaff, and **(D)** grain. Symbols (▲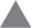) and dashed lines refer to the 2016-2017 growing season, symbols (•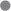) and solid lines refer to 2017-2018 growing season. Black symbols are the control treatment and grey symbols are the in-furrow fertilizer treatment. Only significant (P < 0.05) regressions are shown.

The slopes from the allometric analysis (i.e., log organ *versus* log shoot biomass) plotted against year of release showed no significant trends for leaves (**Figure 5A**). For stem, there was a significant nonlinear relationship during the 2016-2017 growing season, with no changes from 1920 to 1966 and a clear decrease afterwards (**Figure 5B**). The allometric coefficient for chaff increased with year of release in 2016-2017 (**Figure 5C**). The allometric coefficient for grain increased with year of release in both seasons (**Figure 5D**). Fertility treatment only affected the allometric coefficient for stem in 2017-18, when in-furrow fertilizer showed greater slope than control treatment (data not shown).

### Association Between Grain Yield, Crop Traits and Weather Variables

**Figure 6:**
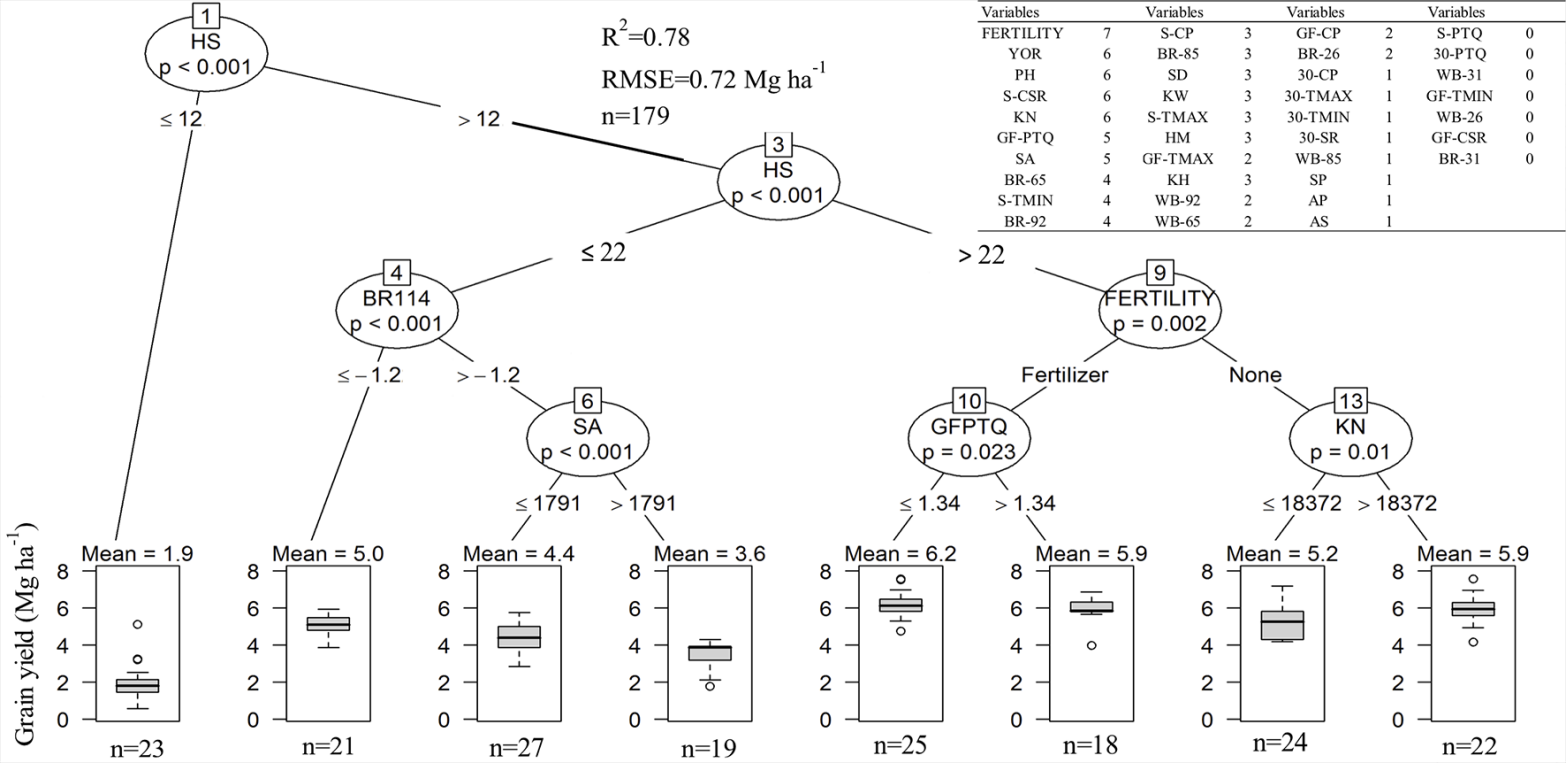
Conditional inference tree for grain yield as related to weather, crop traits, and fertiliser across the entire data set. Boxplots spans first to the third quartile, inside solid line are the means which are also shown above each boxplot. The lower and upper lines show the 5^th^ and 95^th^ percentile, respectively. Inset table shows a list of 37 candidate variables at influencing wheat grain yield and the number of statistical models in which each variable was significantly associated with grain yield, out of a total of seven models. Year of variety release (YOR), plant height (PH), kernel number (KN), head number (HN), stem diameter (SD), kernel weight (KW), head size (HS), thermal time from sowing to anthesis (SA), from sowing to physiological maturity (AP), from anthesis to soft dough (AS), from sowing to physiological maturity (SP), maximum (TMAX) and minimum temperature (TMIN), cumulative solar radiation (CSR), cumulative precipitation (CP), photothermal quotient (PTQ), whole plant biomass (WB), crop biomass rate (BR). Letters left to each variable represent the period, growing season (S), thirty days before anthesis (30), grain filling (GF). Values to the right to each variable represent the growth stage in the Zadoks scale, GS 26, 31, 65, 85, and 92.

In-furrow fertilizer, plant height, year of release, and kernels m^-2^ were positively, and seasonal cumulative solar radiation was negatively associated with grain yield in at least six out of seven models tested (inset table on **Figure 6**). Grain yield related positively with kernel weight, head size, stem diameter, and biomass growth rate between GS 65 and GS 85 in at least half of the models. Seasonal minimum temperature, photothermal quotient during grain filling, timing from sowing to anthesis, and biomass rate at GS 92, were negatively associated with yield. The conditional inference tree suggested that kernels head^-1^ was among the most important determinants of yield, with head size less than 12 kernels resulting in the lowest yields (**Figure 6**). There were significant interactions between head size and biomass rate at GS 92, time from sowing to anthesis, in-furrow fertilizer, and kernel number on wheat yield. The highest yields were attained when heads had more than 22 kernels, in crops with in-furrow fertilizer and with photothermal quotient less than 1.34 MJ m^-2^ d^-1°^C^-1^. In the absence of fertilizer, more kernels m^-2^ related to greater yield.

## Discussion

We evaluated the effects of in-furrow fertilization on grain yield, yield components, and biomass accumulation and partitioning in a set of historical and modern commercial wheat varieties adapted to Kansas, USA. Our results exemplify how direct selection for grain yield changed wheat phenotype during a ~100-year period in a dry subhumid environment. While changes in crop physiological traits in response to breeding have been reported a number of times, to our knowledge, this is the first detailed assessment of changes in biomass partitioning and rates of mass accumulation to different organs as affected by both cultivar and management (i.e., in-furrow fertilizer). Our results can help guide future selection for wheat yield in other dry-environments.

### Plant Height, Stem Diameter, and Phenology

The logistic model suggested a steep decrease in plant height from historic to modern varieties, resulting in two distinct groups. This is a consequence of the introduction of dwarfing genes in modern genotypes (Peng et al., 1999). An optimum wheat plant height between 0.7–1.0 m was described by Richards (1992) in a south-eastern Australian environment, which is shorter than the measurements in the current study. This indicates that there is still scope for shortening wheat varieties in U.S. southern Great Plains. Benefits of shorter varieties include increases in harvest index (Austin et al., 1980; Acreche et al., 2008); standability; and perhaps improvements in grain yield (Donmez et al., 2001; Brancourt-Humel et al., 2003). The logistic model representing changes in stem diameter was not as steep as that for plant height, but historical varieties with thinner stems were more prone to lodging (data not shown). Zuber et al. (1999) and Tripathi et al. (2003) found a strong negative relationship between stem diameter and lodging score. Lodging can decrease the stored photoassimilate reserves (Fischer and Stapper, 1987) and N use efficiency (Brancourt-Humel et al., 2003), reducing grain yield in as much as 35% (Fischer and Stapper, 1987).

The piecewise model suggested a large variation for flowering thermal time in the varieties included in this study between 1920 and 1988, with no substantial changes afterwards. The shorter cycle observed in semidwarf varieties derived from earliness in flowering time but similar or longer duration of grain fill. Earlier flowering associated with reduced shoot biomass at anthesis and the longer grain filling period of modern varieties associated with increased harvest index and yield. Early anthesis has been associated with genetic progress in grain yield of wheat in the U.S. Great Plains (Donmez et al., 2001), in the U.K. (Austin et al., 1980), and Mediterranean environments (Siddique et al., 1989a; De Vita et al., 2007; Giunta et al., 2007). The lack of change in flowering time since 1990s suggests that modern varieties flower at the optimal time for the region, balancing higher risks of spring freeze injury in earlier flowering and greater risks for high temperatures and drought stresses during grain fill with later flowering (Khalil et al., 1995; Sciarresi et al., 2019).

### Grain Yield, N Removal, and Grain Protein Concentration

A sample of winter wheat varieties released between 1920 and 2016 in the U.S. southern Great Plains revealed different rates in yield gain in different time periods, with a small yield gain until ~1970s, accompanied by greater yield gain through ~2000s, and smaller gain afterwards. This small yield progress in recent years was recently reported for a set of commercial varieties from different breeding programs in the region (de Oliveira et al., 2019). Historical sets of wheat varieties have been assessed to estimate the progress of breeding efforts and quantify the impact of management practices (Brancourt-Humel et al., 2003; Acreche et al., 2008; Del Pozo et al., 2014; Lo Valvo et al., 2017; Flohr et al., 2018). In some cases, similar historical trends occurred in different regions (Austin et al., 1980; Cox et al., 1988; Slafer and Andrade, 1989; Sanchez-Garcia et al., 2013; Beche et al., 2014; Lo Valvo et al., 2017; Flohr et al., 2018). The greater yield improvement mid-century was a result of the introduction of the dwarfing genes, which allowed for less lodging (Evenson, 2003).

The trend in yield gain found in this study contrasted with other studies that reported no clear tendency of leveling-off in yield progress (Donmez et al., 2001; Sadras and Lawson, 2011). This divergence might result from the genotype × environment interaction (Sanchez-Garcia et al., 2013), or environmental yield potential might also affect these results, especially when evaluating responses to management (Brancourt-Humel et al., 2003). Finally, the focus of the regional breeding programs may also affect the rate of yield gain (e.g., focusing solely in yield potential *versus* focusing in disease resistance and grain quality) (Fischer and Edmeades, 2010). We also acknowledge that our power to make inferences to changes in phenotype in other regions is relatively low as our data is biased toward varieties developed by a particular wheat-breeding program, and rates of yield gain vary greatly between breeding programs even within a similar geography (Rife et al., 2019). Nonetheless, our analysis offers insights into changes in wheat phenotype in response to breeding for yield in a predominantly dry environment.

Modern varieties removed more N in grain and had lower grain protein concentration than historical ones, suggesting that the decrease in grain protein concentration over time was due to greater improvements in grain yield relative to putative increase in crop nitrogen uptake and/or nitrogen harvest index (Sadras et al. (2016). As expected (Kibite and Evans, 1984; Oury and Godin, 2007; Bogard et al., 2010; Lollato and Edwards, 2015), grain protein concentration declined with grain yield. The decrease in grain protein concentration with higher yield is partially a dilution effect (Kibite and Evans, 1984). Nonetheless, when normalized for yield, grain protein concentration did not change with year of release. This maintenance of protein concentration when corrected for yield, despite substantial increases in grain yield, is likely a response to the emphasis on wheat quality in the region (e.g., mostly bread wheat as opposed to lower quality soft wheat classes) (Baenziger et al., 2001; Fufa et al., 2005).

### Morphological and Physiological Components of Yield Increase

Heads m^-2^ decreased over time in our study, with similar findings reported by Tian et al. (2011) in China. Breeding programs directly selecting for yield in dry environments (e.g., Kansas or the North China Plain) might have indirectly selected for lower tillering and fewer heads per unit area as a soil water conservation strategy (van Herwaarden et al., 1998), perhaps with the exception of dual-purpose (i.e., grazing plus grain) breeding programs for dry regions (Carver et al., 2001). In-furrow fertilizer increased heads m^-2^ by 7%–10%, likely due to greater early-season wheat biomass (Lollato et al., 2013) and tillers plant^-1^ (Sato et al., 1996), increasing heads m^-2^ (Rodríguez et al., 1999). The effects of in-furrow fertilizer increasing heads m^-2^ contrasted with the trends of decreased heads m^-2^ due to breeding. This, in addition to results shown in **Figure 6**, suggests that more heads m^-2^ might not always be desirable in this dry environment, perhaps explaining the inconsistent wheat yields response to in-furrow P in the region (e.g., Lollato et al., 2013; Lollato et al., 2019b).

The increase kernels head^-1^ over time corroborates with findings for other regions (Siddique et al., 1989a; Siddique et al., 1989b; De Vita et al., 2007; Del Pozo et al., 2014). Sanchez-Garcia et al. (2013) reported that the increase in kernels head^-1^ was explained by an increase in spikelets head^-1^ and kernels spikelet^-1^. The introduction of dwarfing genes can partially explain the increase in kernels head^-1^ (Flintham et al., 1997; De Vita et al., 2007), as these genes might favor partitioning of biomass into spikes (Abbate et al., 1998; Miralles et al., 1998), and enhanced survival of floret primordia (Miralles et al., 1998). Interestingly, in-furrow fertilizer reduced kernels per head, perhaps because of the increased number of heads reducing the average head size.

Kernels m^-2^ is considered a coarse-regulator of wheat yield (Slafer et al., 2014). Its progress over decades was reported to relate to improvements in kernels head^-1^ (Slafer and Andrade, 1989; De Vita et al., 2007), head dry weight at anthesis (Acreche et al., 2008), partition of more photoassimilates into the developing heads (Slafer and Andrade, 1989), and growth rate (Sadras and Lawson, 2011). Kernel weight increased from 1920 until 1960s, with no major changes afterwards. While this analysis suggests that selection for yield over time did not change kernel weight (maybe because kernel weight is a fine regulator of wheat yield; Slafer et al., 2014), we note that there were substantial increases in kernels m^-2^ whilst maintaining kernel weight. In-furrow fertilizer decreased average kernel weight, which agrees with Tian et al. (2011). This likely results from more heads formed from later tillers due to in-furrow fertilization.

### Total Biomass, Crop Growth Rate, and Allocation to Plant Components

Most studies comparing historical and modern wheat varieties report biomass at one of few growth stages, more often at physiological maturity (Brancourt-Humel et al., 2003; Giunta et al., 2007; Sadras and Lawson, 2011; Pampana et al., 2013; Wang et al., 2017). Only a handful of studies reported dynamics of shoot biomass more times in the season (e.g., Austin et al., 1980; Siddique et al., 1989a; Shearman et al., 2005; Acreche et al., 2008; Pampana et al., 2013; Flohr et al., 2018).

The similarity among wheat varieties in total biomass and initial growth rate suggests that the chronological changes in biomass accumulation responsible for greater grain yield occurred later in the season. At anthesis, tall varieties had greater total biomass, partially due to the longer period required to reach this growth stage as compared to shorter-cycled semidwarf varieties (Álvaro et al., 2008; Flohr et al., 2018). Despite a greater biomass, its partitioning into reproductive organs was less efficient in tall varieties, as the allometric coefficient consistently increased when related to varieties’ year of release. Reports by Slafer et al. (1990) and Álvaro et al. (2008) agreed with our findings and showed that biomass partitioning to the chaff in wheat varieties increased over time. The same levels of biomass with greater HI in semidwarf varieties and the consistent increase in allometric coefficient for grain versus year of release across all site-years suggests that yield increases in modern wheat varieties resulted from more efficient partitioning of assimilates to the grains rather than greater biomass, possibly due to greater remobilization (Pampana et al., 2013). Likewise, previous studies have reported no substantial changes in biomass accumulation at maturity over the years (Austin et al., 1980; Calderini et al., 1995; Royo et al., 2007; Acreche et al., 2008; Kitonyo et al., 2017). The inconsistent results in allometric exponents for the different years of this study for leaves, chaff, and stem might result from different patterns of accumulation and remobilization of dry matter as affected by environment, similar to the results of Pampana et al. (2013).

Harvest index has been associated with genetic yield gain in wheat (Slafer and Andrade, 1989; Royo et al., 2007; Zhou et al., 2007). However, Austin et al. (1980) proposed that theoretical biological limit for harvest index in well-watered crops was ~0.62, suggesting that there might have room for further improvement in modern hard red winter wheat varieties in the study region (i.e., harvest index ~0.44 for semidwarf varieties), although this limit might be lower in dryland environments. Tall and semidwarf varieties at maturity presented similar shoot biomass, suggesting that improvements in grain yield over time resulted from a greater ability of semidwarf varieties to allocate assimilates to the grain (Tian et al., 2011).

## Conclusions

Kansas winter wheat varieties increased grain yield over time, but there was a decrease on the pace of progress after 1990s. Selection for yield increased kernels per area and kernels per head in modern semidwarf cultivars. Semidwarf varieties also flowered earlier than tall varieties and had longer grain-filling period, which associated with less biomass at anthesis and greater harvest index, respectively. Increases in allometric coefficient with year of release also suggested that greater yield in semidwarf cultivars resulted from a greater ability to allocate dry matter into the grain even at similar shoot mass. The decrease in grain protein concentration over time was solely a function of increases in grain yield, as there was no relationship between the residuals of grain protein concentration and grain yield *versus* year of release. While in-furrow fertilizer increased biomass and grain yield, the lack of interaction suggests that semidwarf varieties were not more responsive than tall varieties to in-furrow fertilizer when otherwise well fertilized (i.e., all plots received enough N for a 6 Mg ha^-1^ yield goal).

## Data Availability Statement

The datasets generated for this study are available on request to the corresponding author.

## Author Contributions

The manuscript was reviewed and approved for publication by all authors. RL and AF conceived and designed the field experiment. RM collected data, analyzed the data, and drafted the manuscript. VS, IC, and RL made substantial contributions to data analysis and interpretation, and manuscript editing. AF and DD edited the manuscript.

## Funding

This project was partially sponsored by the Kansas Wheat Commission (Award Number PP34916) and The Mosaic Company.

## Conflict of Interest

The authors declare that the research was conducted in the absence of any commercial or financial relationships that could be construed as a potential conflict of interest.

## Footnotes

1 Imidacloprid: 1-[(6-Chloro-3-pyridinyl) methyl]-N-nitro- 2-imidazolidinimine), 0.95 ml 100 kg seed^-1^ of metalaxyl: N-(2,6-dimethylphenyl)-N-(methoxyacetyl) alanine methyl ester.

2 Tebuconazole: alpha-[2-(4-chlorophenyl) ethyl]-alpha- (1,1-dimethyl-ethyl)-1H-1,2,4-triazole-1- ethanol).

3 Picoxystrobin (Methyl (∝E)- ∝-(methoxymethylene)-2-[[[6-(trifluoromethyl)-2 pyridinyl]oxy]methyl]benzeneacetate.

4 Cyproconazole ∝-(4-chlorophenyl)- ∝-(1-cyclopropylethyl)-1H-1,2,4-triazole-1-ethanol.
