# Supplementary material for: Changes in the Phenotype of Winter Wheat Varieties Released Between 1920 and 2016 in Response to In-Furrow Fertilizer: Biomass Allocation, Yield, and Grain Protein Concentration
Source: Front Plant Sci. 2020 Jan 30;10:1786. doi: 10.3389/fpls.2019.01786 (PMC7002544; doi:10.3389/fpls.2019.01786)
Supplement: Supplementary file 1 [file DataSheet_1.docx]

**Supplement Table 1.** *Similarity matrix for eight winter wheat varieties using genotyping-by-sequencing (GBS) single nucleotide polymorphisms (SNPs). A total of 9946 SNPs were used for the analysis to determine the percent*

| Varieties  (Year of release) | Kharkoff (1920) | Scout66 (1966) | Karl92 (1988) | Jagger (1994) | Jagalene (2001) | Fuller (2006) | KanMark (2014) | Larry (2016) |
| --- | --- | --- | --- | --- | --- | --- | --- | --- |
| Kharkoff (1920) |  | 0.86 | 0.58 | 0.41 | 0.52 | 0.49 | 0.54 | 0.54 |
| Scout66 (1966) |  |  | 0.51 | 0.38 | 0.47 | 0.45 | 0.50 | 0.50 |
| Karl92 (1988) |  |  |  | 0.34 | 0.45 | 0.52 | 0.61 | 0.63 |
| Jagger (1994) |  |  |  |  | 0.78 | 0.75 | 0.37 | 0.51 |
| Jagalene (2001) |  |  |  |  |  | 0.63 | 0.51 | 0.52 |
| Fuller (2006) |  |  |  |  |  |  | 0.42 | 0.48 |
| KanMark (2014) |  |  |  |  |  |  |  | 0.52 |
| Larry (2016) |  |  |  |  |  |  |  |  |

**Supplement Table 2.** Significance of variety, fertility, and variety × fertility interaction derived from the analysis of variance for grain yield, grain-N removal, grain protein concentration (GPC), head number (HN), head size (HS), kernel number (KN), kernel weigh (KW), harvest index (HI), plant height (PH), stem diameter (SD), volume weight, biomass and crop growth rate at GS 26, 31, 65, 85, and 92, biomass by plant component at GS 26, 31, 65, 85, and 92 for leaves (L), stem (S), chaff (C), and grains (G).

|  | 2016-17 |  |  | 2017-18 |  |  |
| --- | --- | --- | --- | --- | --- | --- |
| Variables | Variety | Fertility | Variety x Fertility | Variety | Fertility | Variety x Fertility |
| Grain yield | <.001 | 0.033 | 0.069 | <.001 | <.001 | 0.776 |
| Grain-N removal | <.001 | 0.091 | 0.189 | <.001 | 0.007 | 0.823 |
| GPC | <.001 | 0.151 | 0.026 | <.001 | 0.034 | 0.302 |
| HN | 0.001 | 0.003 | 0.569 | 0.009 | 0.002 | 0.859 |
| HS | <.001 | 0.002 | 0.938 | <.001 | 0.001 | 0.845 |
| KN | <.001 | 0.761 | 0.878 | <.001 | 0.685 | 0.999 |
| KW | <.001 | 0.023 | 0.521 | <.001 | 0.001 | 0.117 |
| HI | <.001 | 0.881 | 0.532 | 0.009 | 0.597 | 0.981 |
| PH | <.001 | 0.069 | 0.511 | <.001 | 0.859 | 0.761 |
| SD | <.001 | 0.597 | 0.917 | 0.252 | 0.499 | 0.101 |
| Volume weight | <.001 | 0.033 | 0.395 | <.001 | 0.003 | 0.128 |
| GS 26 | 0.002 / 0.002 | <.001 / <.001 | 0.607 / 0.495 | 0.502 / 0.434 | <.001 / <.001 | 0.667 / 0.638 |
| GS 31 | 0.727 / 0.791 | <.001 / <.001 | 0.697 / 0.679 | 0.106 / 0.074 | <.001 / <.001 | 0.831 / 0.808 |
| GS 65 | <.001 / 0.276 | <.001 / 0.040 | 0.044 / 0.163 | <.001 / 0.369 | <.001 / 0.007 | 0.842 / 0.645 |
| GS 85 | 0.001 / <.001 | 0.001 / 0.067 | 0.431 / 0.778 | 0.846 / 0.347 | <.001 / 0.059 | 0.372 / 0.479 |
| GS 92 | 0.731 / 0.002 | 0.169 / 0.071 | 0.818 / 0.799 | 0.180 / 0.978 | 0.039 / 0.005 | 0.988 / 0.925 |
| GS 26 - L | 0.002 | <.001 | 0.607 | 0.502 | <.001 | 0.667 |
| GS 31 -L | 0.398 | <.001 | 0.725 | 0.306 | <.001 | 0.987 |
| GS 31 - S | 0.016 | <.001 | 0.531 | 0.007 | <.001 | 0.285 |
| GS 65 - L | 0.049 | <.001 | 0.481 | 0.055 | <.001 | 0.723 |
| GS 65 - S | <.001 | <.001 | 0.02 | <.001 | <.001 | 0.418 |
| GS 65 - C | 0.003 | 0.005 | 0.034 | 0.004 | 0.001 | 0.582 |
| GS 85 - L | 0.105 | <.001 | 0.069 | 0.182 | <.001 | 0.359 |
| GS 85 - S | 0.005 | 0.002 | 0.579 | <.001 | <.001 | 0.055 |
| GS 85 -C | <.001 | 0.001 | 0.225 | 0.32 | <.001 | 0.577 |
| GS 85 - G | <.001 | 0.068 | 0.463 | <.001 | 0.009 | 0.815 |
| GS 92 -L | 0.009 | <.001 | 0.642 | 0.054 | 0.002 | 0.948 |
| GS 92 -S | <.001 | 0.007 | 0.568 | <.001 | 0.004 | 0.62 |
| GS 92 -C | 0.009 | 0.344 | 0.854 | 0.582 | 0.048 | 0.908 |
| GS 92 - G | <.001 | 0.334 | 0.926 | <.001 | 0.459 | 0.998 |

**Supplement Table 3.** *Shoot biomass and crop growth rate in whole plant at Zadoks 26, 31, 65, 85 and 92 of wheat varieties released between 1920 and 2016, fertilizer treatment, and their interaction during the growing seasons 2016-17 and 2017-18. Variety and fertilizer means were averaged across locations within growing season.*

| Year | Variety (Year of release) | Fertilizer | GS 26 (g m^2^ / g m^2^ GDD ⁰C^-1^) | GS 31 (g m^2^ / g m^2^ GDD ⁰C^-1^) | GS 65 (g m^2^ / g m^2^ GDD ⁰C^-1^) | GS 85 (g m^2^ / g m^2^  GDD ⁰C^-1^) | | GS 92 (g m^2^ / g m^2^  GDD ⁰C^-1^) | |  |
| --- | --- | --- | --- | --- | --- | --- | --- | --- | --- | --- |
| 2016-17 | Kharkof (1920) |  | 61a / 0.095a | 247 / 0.267 | 1108a / 1.250 | | 1372c / 0.536b | | 1421 / 0.384ab | |
|  | Scout 66 (1966) |  | 57a / 0.089a | 285 / 0.328 | 1067ab / 1.388 | | 1358c / 0.424b | | 1518 / 1.269a | |
|  | Karl 92 (1988) |  | 59a / 0.092a | 267 / 0.299 | 816c / 1.443 | | 1537bc / 1.131a | | 1348 / -0.674bc | |
|  | Jagalene (2001) |  | 54a / 0.085a | 253 / 0.286 | 989b / 1.474 | | 1889a / 1.419a | | 1530 / -1.814c | |
|  | Fuller (2006) |  | 43b / 0.069b | 248 / 0.294 | 858c / 1.468 | | 1747ab / 1.424a | | 1446 / -1.065bc | |
|  | KanMark (2014) |  | 43b / 0.069b | 239 / 0.281 | 801c / 1.355 | | 1604bc / 1.150 a | | 1447 / -0.668bc | |
|  | Larry (2016) |  | 58a / 0.091a | 252 / 0.278 | 976b / 1.452 | | 1749ab / 1.254a | | 1441 / -1.487c | |
|  |  | In-furrow | 64A / 0.099A | 290A / 0.326A | 1005A / 1.451A | | 1722A / 1.128 | | 1492 / -0.923 | |
|  |  | Control | 43B / 0.069B | 221B / 0.255B | 885B / 1.358B | | 1494B / 0.968 | | 1408 / -0.236 | |
| 2017-18 | Kharkof (1920) |  | 36 / 0.061 | 157 / 0.178 | 841a / 1.212 | | 1103 / 0.592 | | 1033 / -0.263 | |
|  | Scout 66 (1966) |  | 30 / 0.052 | 199 / 0.249 | 880a / 1.371 | | 1193 / 0.591 | | 1133 / -0.358 | |
|  | Karl 92 (1988) |  | 39 / 0.067 | 167 / 0.190 | 621b / 1.350 | | 1091 / 0.759 | | 1011 / -0.559 | |
|  | Jagger (1994) |  | 35 / 0.61 | 170 / 0.200 | 651b / 1.319 | | 1168 / 0.858 | | 1174 / -0.367 | |
|  | Jagalene (2001) |  | 36 / 0.062 | 165 / 0.190 | 679b / 1.292 | | 1128 / 0.792 | | 1116 / -0.098 | |
|  | Fuller (2006) |  | 32 / 0.055 | 151 / 0.175 | 624b / 1.338 | | 1086 / 0.777 | | 1030 / -0.304 | |
|  | KanMark (2014) |  | 34 / 0.059 | 169 / 0.200 | 716b / 1.493 | | 1174 / 0.761 | | 1179 / 0.024 | |
|  | Larry (2016) |  | 34 / 0.058 | 138 / 0.153 | 657b / 1.428 | | 1164 / 0.843 | | 1136 / -0.181 | |
|  |  | In-furrow | 42A / 0.071A | 200A / 0.234A | 773A / 1.422A | | 1233A / 0.806 | | 1134A / -0.669B | |
|  |  | Control | 27B / 0.047B | 129B / 0.150B | 644B / 1.279B | | 1044B / 0.688 | | 1069B / 0.142A | |

Values followed by the same letter within growing and treatment season are not statistically different at α = 0.05.
Jagger was not included in the 2016-17 growing season analysis
